# Reasons for Discontinuation or Change of Selective Serotonin Reuptake Inhibitors in Online Drug Reviews

**DOI:** 10.1001/jamanetworkopen.2023.23746

**Published:** 2023-07-17

**Authors:** Su Golder, Dominique Medaglio, Karen O’Connor, Sean Hennessy, Robert Gross, Graciela Gonzalez Hernandez

**Affiliations:** 1 Department of Health Sciences, University of York, York, United Kingdom; 2 Department of Biostatistics, Epidemiology and Informatics, Perelman School of Medicine, University of Pennsylvania, Philadelphia; 3 Department of Computational Biomedicine, Cedars-Sinai Medical Center, West Hollywood, California

## Abstract

**Importance:**

Selective serotonin reuptake inhibitors (SSRIs) are a commonly prescribed medication class to treat a variety of mental disorders. However, adherence to SSRIs is low, and uncovering the reasons for discontinuation among SSRI users is an important first step to improving medication persistence.

**Objective:**

To identify the reasons SSRIs are discontinued or changed, as reported by patients and caregivers in online drug reviews.

**Design, Setting, and Participants:**

This qualitative study used natural language processing and machine learning to extract mentions of changes in SSRI intake from 667 drug reviews posted on the online health forum WebMD from September 1, 2007, to August 31, 2021. The type of medication change, including discontinuation, switch to another medication, or dose change and the reason for the change were manually annotated. In each instance in which an adverse event was reported, the event was categorized using Medical Dictionary for Regulatory Activities primary system organ class (SOC) codes, and its relative frequency was compared with that in spontaneous reporting systems maintained by the US Food and Drug Administration and the UK Medicines and Healthcare Products Regulatory Agency.

**Main Outcomes and Measures:**

Reasons for SSRI medication change as assessed using SOC codes.

**Results:**

In total, 667 reviews posted by 659 patients or caregivers (516 [78%] of patients were female; 410 [62%] 25-54 years of age) were identified that indicated a medication change: 335 posts indicated SSRI discontinuation, 188 posts indicated dose change, and 179 posts indicated switched medications. Most authors 625 (95%) were patients. The most common reason for medication discontinuation or switching was adverse events experienced, and the most common reason for dose change was titration. Both uptitration and downtitration were initiated by either a health care professional or patient. The most common adverse events were classified by SOC codes as psychiatric disorders, including insomnia, loss of libido, and anxiety. Compared with those in regulatory data, psychiatric adverse events, adverse events recorded by investigations (mostly weight gain) and adverse events associated with the reproductive system (mostly erectile dysfunction) were reported disproportionately more often.

**Conclusions and Relevance:**

This qualitative study of online drug reviews found that useful information was provided directly by patients or their caregivers regarding their medication behavior, specifically, information regarding SSRI treatment changes that may inform interventions to improve adherence. These findings suggest that these reported adverse events may be associated with SSRI persistence and that people may feel more inclined to report such events on social media than to clinicians or regulatory agencies.

## Key Points

**Question:**

What reasons for changes in the use of selective serotonin reuptake inhibitors (SSRIs) are reported on a popular health website?

**Findings:**

This qualitative study of 667 online drug reviews found that the most common reason for discontinuing SSRI use or switching to another SSRI was adverse events experienced, and the most common reason for dose change was titration. Adverse events categorized under psychiatric disorders (mostly apathy, anxiety, insomnia, and loss of libido), investigation results (mostly weight gain), and reproductive system and breast disorders (mostly sexual dysfunction) appeared disproportionately more often in online drug reviews than in US and UK regulatory adverse event reporting data.

**Meaning:**

These results suggest that reasons for changes in SSRI use can be identified in online drug reviews and that adverse events mentioned may reflect those more salient to patients for discontinuing their medication.

## Teaser

This qualitative study describes the reasons for selective serotonin reuptake inhibitor changes as reported in comments and reviews on the online health forum WebMD by patients and caregivers.

## Introduction

Mental disorders, including depression and anxiety, are a leading cause of global disease burden, affecting 1 in 8 people worldwide.^1^ The health, economic, and social effects of mental disorders are substantial, resulting in increased morbidity and mortality, loss of productivity, and increased discrimination and stigma.^1,2,3^ Antidepressant medications are widely used in the treatment of many mental disorders, with selective serotonin reuptake inhibitors (SSRIs) among the most prescribed treatments. Long-term adherence to SSRIs is important for the remission of symptoms and prevention of relapse,^4^ yet nonadherence to SSRIs is common.

Medication adherence can be described as 3 different phases of behaviors: initiation, implementation over time, and discontinuation.^5^ Early discontinuation, or nonpersistence, is common with antidepressants, with approximately 25% of patients discontinuing treatment within 1 month and as many as 68% of patients within 3 months.^6^ The reasons for SSRI nonadherence have been widely studied in clinical settings. Generally, self-reported reasons for nonadherence to antidepressants are varied and can differ by each SSRI. Examples include forgetfulness; thinking the medication was unnecessary; delayed onset of action; and adverse effects, including dry mouth, dizziness, sleep issues, and sexual dysfunction.^4,7^ Discontinuation of SSRIs without clinician guidance is of particular concern as patients can experience new symptoms,^8^ the return of their original symptoms,^8^ and SSRI withdrawal syndrome.^9,10,11^

Efforts to improve antidepressant persistence have yielded limited success,^6^ often impeded by an incomplete understanding of the complex barriers that patients face in the real world.^12^ Understanding the reasons behind medication nonpersistence is paramount to the design and implementation of future adherence interventions.^13,14^ In traditional research involving surveys or interviews, patients may withhold information about their medication behaviors,^15,16^ and some subpopulations may be underrepresented.^17^ Online drug reviews provide a new opportunity to gather large numbers of first-hand reports and experiences of medications^18,19,20^ and complement other information sources.^19,20,21,22^ To date, there has not been a full investigation of SSRI user drug reviews. The objective of this study was to describe the reasons why SSRIs are discontinued or changed, as reported by patients and caregivers in online comments and reviews.

## Methods

We conducted a retrospective qualitative study to examine reviews of SSRIs on an American health information website, WebMD.^23^ The site provides open access to anonymous drug reviews posted by the public. Each review is assigned to a named drug and has 1- to 5- star ratings evaluating satisfaction, effectiveness, and ease of use (with 1 star being the lowest) and a free-text comment section allowing users to describe their experience. The institutional review boards of the University of York, UK, the University of Pennsylvania, Philadelphia, and Cedars-Sinai Medical Center, West Hollywood, California, deemed this research exempt from review because all data used in the study are publicly available and reported in aggregate with no identifiable data presented. This study followed the Standards for Reporting Qualitative Research (SRQR) reporting guideline.

### Data Set

Our website data set comprised 343 459 reviews for all drugs posted from September 1, 2007, to August 31, 2021.^24^ This data set represented all reviews posted at the time of collection. We gathered all available data points for each review, including the free-text written review, as well as the information in structured fields: date posted, patient age, patient sex, time taking the drug, author role (patient or caregiver), medical condition, overall rating, and ratings for effectiveness, ease of use, and satisfaction. All data were collected according to the website’s terms of use and were publicly available at the time of collection and analysis.

Prior to this study, our group developed a machine learning pipeline to analyze WebMD reviews^24,25^ for discussion of changes in medication. Briefly, the pipeline consisted of 2 modules: a classification module and a sequence labeler. The classification module used a deep neural network with bidirectional encoder representations from transformer (BERT)-based^26^ contextual embeddings as inputs to estimate the probability that the free-text review contains a mention of changing or discontinuing a medication. This module was fine-tuned using 12 972 annotated reviews. Reviews categorized as positive for medication change were then processed by the sequence labeler module, which was designed using BERT-based^26^ contextual embeddings, to extract the specific reasons mentioned for changing or discontinuing the medication. Our classifier module achieved good performance (F1 score, 0.874), whereas the sequence labeler showed moderate performance (F1 score, 0.696). All collected reviews underwent processing using our pipeline.

### Automatic and Manual Extraction

From the collection of reviews processed through our pipeline, we selected reviews of 7 SSRI medications—escitalopram, sertraline, citalopram, paroxetine, fluvoxamine, vortioxetine, and fluoxetine—denoted by their generic or common brand names to study reasons for medication change with SSRIs. Based on an assessment of available time and resources, we determined that 1000 reviews, classified as discussing medication change, would be manually reviewed. Given the low frequency of fluvoxamine and vortioxetine reviews that described a medication change (Table 1), all reviews for these 2 medications were retained for analysis. For the remaining SSRIs, a random sample of reviews was selected, proportional to their frequency. We used automated extracted reasons for change to help manually annotate the reviews.

**Table 1.** Selective Serotonin Receptor Inhibitors With Reviews on a Health-Related Website

Generic SSRI name	No. of reviews^a^	Reviews classified as describing a medication change, No. (%)	No. of reviews manually reviewed	No. of reviews analyzed	No. of medication changes
Escitalopram	4343	1962 (45)	215	147	153
Sertraline	3190	1421 (45)	156	120	127
Citalopram	3803	1418 (37)	155	92	103
Paroxetine	1925	989 (51)	108	80	84
Fluvoxamine	253	121 (48)	121	72	72
Vortioxetine	276	149 (54)	149	88	88
Fluoxetine	2318	924 (40)	96	68	76
Total	16 108^a^	6984 (43)	1000	667	703

Abbreviation: SSRI, selective serotonin reuptake inhibitor.

^a^ In total, 2128 reviews had no written comment.

For each of the 1000 reviews, 2 of us (S.G. and D.M.) checked whether the review was a true mention of a medication change. Changes were further classified as a medication switch (if the patient discussed taking a different SSRI or another antidepressant after stopping the reviewed drug), a change in medication dose (which included increases and decreases in dose, such as titrations), or a discontinuation (if the patient did not mention taking a new antidepressant after stopping the reviewed drug). We extracted data on the stated reasons for a dose change, medication switch or discontinuation. When adverse events were reported as the reason for medication change, the adverse event terms were standardized using the Medical Dictionary for Regulatory Activities (MedDRA) preferred term codes. To facilitate comparison with other sources, the preferred term codes were assigned to 1 of 27 MedDRA broader categories of primary system organ class (SOC) codes (eg, psychiatric disorders; reproductive system and breast disorders; and injury, poisoning, and procedural complications). For those with multiple paths, the primary SOC code was selected. Many SOC code categories contain the term “disorders,” although we acknowledge that adverse events are rarely disorders. In addition, the SOC code “investigations” is used for test results and includes weight, hormone, and lipid analyses. We collected data on the days from starting the medication until an adverse event occurrence, whether the adverse event stopped after drug discontinuation, whether the patient restarted the drug and, if so, whether the adverse event recurred. We categorized withdrawal symptoms separately from adverse events.

In addition, we noted instances of commonly mentioned themes using qualitative content analysis^27^ with an inductive approach.^25^ We created an annotation guide (eAppendix in Supplement 1) based on themes unique to SSRIs. Due to the small numbers of reviewers describing any 1 theme (<6%), we did not investigate any themes relating to different characteristics or behaviors of the people posting.

Although it is acknowledged that differences in the adverse events reported to spontaneous reporting systems and on social media may result from different collection methods, data from spontaneous reporting systems are still the best available data source for comparison.^28^ We therefore collected data from the US Food and Drug Administration Adverse Event Reporting System (FAERS) and the UK Medicines and Healthcare Products Regulatory Agency (MHRA) databases from inception to September 30, 2022, and categorized them using the same MedDRA SOC codes for comparison.

## Results

In total, 16 108 SSRI reviews were collected, and 6984 were classified through our pipeline as discussing an SSRI medication change. A subset of 1000 unique reviews was manually reviewed, of which 810 were verified as true positives for mentioning a medication change. We excluded 143 reviews from further analysis because the change detected in the review was not relevant to our study, including discussion of change for other medications, changes in the timing of taking the medication, nonadherence (eg, missing or forgetting doses), and switching from a non-SSRI to the SSRI reviewed. The remaining 667 reviews discussed at least 1 SSRI medication change (including dose change, discontinuation, and medication switch) (Table 1). In total, 703 SSRI medication changes were contained in 667 reviews.

### Characteristics of Authors

Eight authors posted more than 1 review (for different SSRIs); thus, 659 people were included in the analysis. Overall, SSRI users were predominately female (516 [78%]), and this pattern persisted for each SSRI (range, 52 of 72 [72%] for fluvoxamine to 78 of 92 [85%] for citalopram). Most authors were patients (625 [95%]), with only 13 caregivers (2%), and 21 were unknown (missing data). Most users (410 [62%]) were 25 to 54 years of age (eFigure 1 in Supplement 1), and 380 patients (58%) received SSRIs for treatment of a form of depression (eTable 1 in Supplement 1).

### Medication Duration and Satisfaction

Most authors reported the medication intake duration as less than 6 months (eFigure 2 in Supplement 1). The mean (SD) overall ratings for the medications on a scale of 1 to 5 (with 5 being best) was 3.3 (1.3) for overall satisfaction, 3.9 (1.4) for ease of use, 3.1 (1.6) for effectiveness, and 2.7 (1.6) for satisfaction.

### Reasons for Change

The most common medication change was discontinuation (335 posts), followed by dose change (188 posts) and switching medication (179 posts). Most reviews gave 1 reason for medication change (679 of 703 [97%]). The most common reason given for medication change was adverse events experienced (346), followed by titration (medication change only, 143), lack of effectiveness (98), felt it was not necessary (25), or other (eg, pregnancy or ran out of the medication; 13) (eTable 2 in Supplement 1). In the 188 posts for medication change, 122 indicated an increased dose, 44 a reduced dose, 17 both increased and decreased dose, and 5 with unknown direction change (eg, “I changed my dose”).

### Health Care Professional Involvement

Some posts described not consulting with health care professionals or even going against their advice (eTable 3 in Supplement 1). While a dose increase or switching medication may require the involvement of a health care professional, a dose reduction or drug discontinuation could be undertaken without the involvement of a health care professional. Of 335 posts indicating the discontinuation of an SSRI, 62 (19%) did so without consulting a health care professional, and 36 (11%) did so with consulting a health care professional, but whether a health care professional was consulted was unclear in most instances (237 [71%]).

Of authors noting a reduction in dose, 8 of 44 (18%) did so without consulting a health care professional, 10 of 44 (23%) did so with consulting a health care professional, but in most instances (26 of 44 [59%]) it was unclear.

### Adverse Events

The most common primary MedDRA SOC codes were psychiatric disorders, followed by nervous system disorders and gastrointestinal disorders (Table 2). Only 60 posts mentioned the time to adverse event occurrence (median, 1 day; range, 0-180 days), and only 60 posts described a rechallenge, mostly without stating if the adverse events returned (54 cases).

**Table 2.** Primary Medical Dictionary for Regulatory Activities System Organ Class Codes for Adverse Events Leading to Medication Changes

Primary SOC code	No. of occurrences mentioned^a^	Common examples
Psychiatric disorders	339	Insomnia, loss of libido, mood disorders, suicidal ideation, anxiety
Nervous system disorders	161	Dizziness, headache, somnolence
Gastrointestinal disorders	124	Abdominal discomfort, diarrhea, and vomiting
Investigations	103	Weight gain
General disorders and administration site conditions	79	Fatigue, malaise
Skin and subcutaneous tissue disorders	50	Pruritus, hyperhidrosis
Reproductive system and breast disorders	39	Sexual dysfunction
Metabolism and nutrition disorders	21	Increased or decreased appetite
Musculoskeletal and connective tissue disorders	13	Muscle spasms, muscle aches or weakness
Respiratory, thoracic, and mediastinal disorders	11	Excessive yawning, sinus congestion
Cardiac disorders	8	Palpitations
Eye disorders	12	Blurred vision, vision impairment, and eye pain
Renal and urinary disorders	5	Frequent urination, loss of bladder control
Ear and labyrinth disorders	2	Hearing impairment, tinnitus
Injury, poisoning, and procedural complications	1	Bruising

^a^ In addition to the 968 adverse events given, 69 users mentioned experiencing withdrawal symptoms; some users experienced more than 1 adverse event.

Discontinuation was the most common reason for medication change across all 7 SSRIs (Table 3), and psychiatric disorders and nervous system disorders adverse events featured highly in all SSRI drug reviews.

**Table 3.** Medication Change, Satisfaction, and Top Primary System Organ Class Codes for Adverse Events Reported for 7 Selective Serotonin Reuptake Inhibitors on a Health-Related Website

Item	Escitalopram	Sertraline	Citalopram	Paroxetine	Fluvoxamine	Vortioxetine	Fluoxetine
No. of medication changes	153	127	103	84	72	88	76
No. of AEs	246	183	126	116	101	157	90
Discontinuation, No. (%)	82 of 153 (54)	54 of 127 (43)	42 of 103 (41)	41 of 84 (49)	33 of 72 (46)	46 of 88 (52)	37 of 76 (49)
Switch, No. (%)	33 of 153 (22)	39 of 127 (31)	29 of 103 (28)	26 of 84 (31)	12 of 72 (17)	18 of 88 (20)	22 of 7 (29)
Dose change, No. (%)	38 of 153 (25)	34 of 127 (27)	32 of 103 (31)	17 of 84 (20)	27 of 72 (38)	24 of 88 (27)	17 of 76 (22)
Overall satisfaction, mean (SD)^a^	3.3 (1.3)	3.3 (1.3)	3.4 (1.4)	3.4 (1.3)	3.4 (1.3)	2.7 (1.4)	3.3 (1.3)
Effectiveness, mean (SD)^a^	3.1 (1.6)	3.2 (1.5)	3.3 (1.6)	3.5 (1.6)	3.3 (1.5)	2.5 (1.6)	3.1 (1.5)
Ease of use, mean (SD)^a^	4.1 (1.3)	4.0 (1.4)	4.1 (1.4)	3.8 (1.3)	4.0 (1.3)	3.3 (1.7)	4.0 (1.4)
Satisfaction, mean (SD)^a^	2.6 (1.6)	2.7 (1.5)	2.9 (1.6)	2.8 (1.6)	2.9 (1.6)	2.1 (1.5)	2.9 (1.6)
First SOC ranking (No. of reviews)^b^	Psychiatric disorders (69)	Psychiatric disorders (83)	Psychiatric disorders (36)	Psychiatric disorders (34)	Psychiatric disorders (36)	Gastrointestinal disorders (46)	Psychiatric disorders (37)
Second SOC ranking (No. of reviews)^b^	Nervous system disorders (46)	Investigations (20)	Nervous system disorders (22)	Nervous system disorders (24)	Nervous system disorders (18)	Psychiatric disorders (44)	Nervous system disorders (14)
Third SOC ranking (No. of reviews)^b^	General disorder and administration site conditions (30)	Gastrointestinal disorders (20)	Gastrointestinal disorders (14)	Investigations (24)	General disorders and administration site conditions (11)	Nervous system disorders (21)	Investigations (10)
Fourth SOC ranking (No. of reviews)^b^	Investigations (27)	Nervous system disorders (16)	Investigations (7)	Reproductive system and breast disorders (8)	Gastrointestinal disorders(9)	General disorders and administration site conditions(9)	Gastrointestinal disorders (7); general disorders and administration site conditions (7)

Abbreviations: AEs, adverse events; SOC, system organ class; SSRI, selective serotonin receptor inhibitor.

^a^ Rank 1 to 5 stars, with 1 being the lowest rating.

^b^ Limited to the top 4 SOC rankings due to the small numbers of AEs beyond that point in each category when restricted to specific SSRI medication.

### Comparison With Spontaneous Reporting Systems

Data from FAERS and MHRA were in general agreement with our data from online reviews (Figure). The category reproductive system and breast disorders was among the top 4 adverse events in individual SSRI data from the website but not in FAERS, and injury, poisoning, and procedural complications was in the top 4 in FAERS but not in health website data (Table 3; eTable 4 in Supplement 1).

**Figure.**
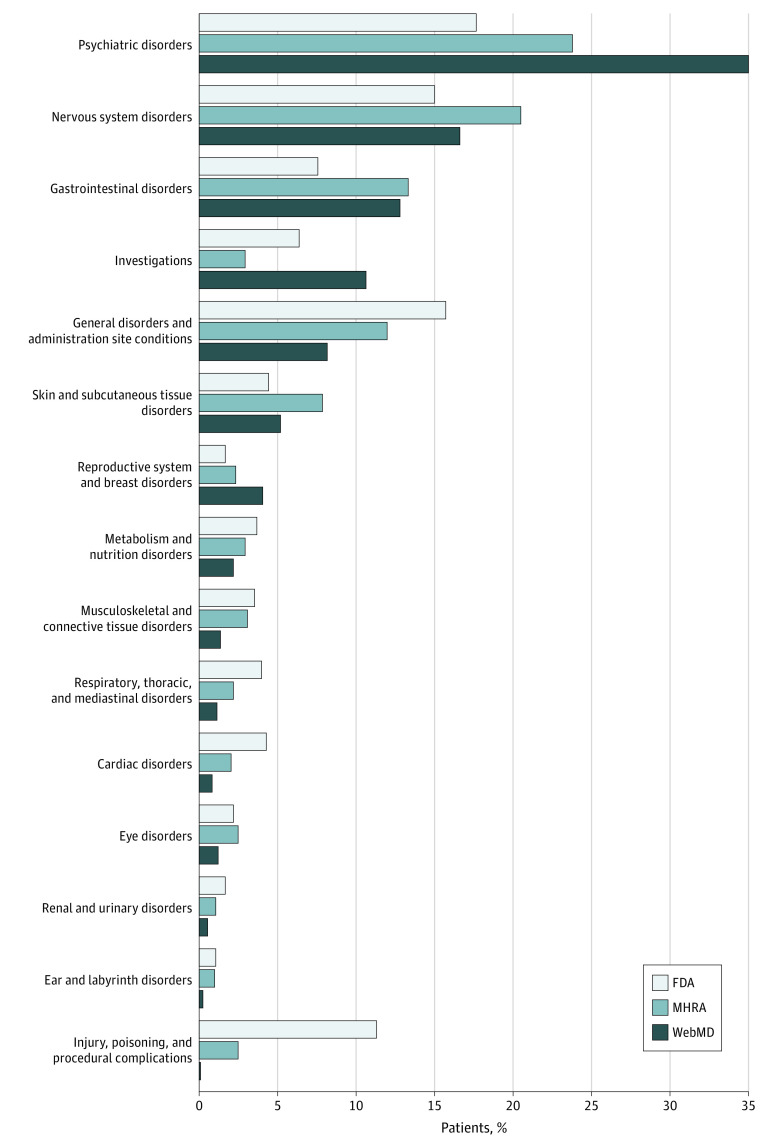
Percentage of Adverse Events Reported in Website Reviews and US Food and Drug Administration (FDA) Adverse Event Reporting System and UK Medicines and Healthcare Products Regulatory Agency (MHRA) Databases for Each Medical Dictionary for Regulatory Activities Primary System Organ Class Code

We calculated the difference in adverse events reported (in percentage points) between FAERS and the health website and found that in most instances there was not a marked difference (eTable 5 in Supplement 1). However, whereas injury, poisoning, and procedural complications (difference, −11.2 percentage points) and general disorders and administration site conditions (difference, −7.5 percentage points) appeared less frequently in the data from the health website than FAERS data, the code psychiatric disorders was more prevalent on the website (difference, 17.3 percentage points). The most MedDRA subclasses of psychiatric disorders experienced were mood disorders and disturbances (64 people posting), particularly apathy; sleep disorders and disturbances (57 people posting), particularly insomnia; anxiety disorders and symptoms (48 people posting); and sexual dysfunctions, disturbances, and gender identity disorders (38 people posting), primarily loss of libido.

We also found general agreement between data from the health website and MHRA data. However, a comparison of the top 4 rankings found reproductive system and breast disorders and investigations in website data but not MHRA and skin and subcutaneous tissue disorders in MHRA but not website data (Table 3; eTable 6 in Supplement 1). The differences in percentage points did not differ substantially between MHRA and the website (eTable 7 in Supplement 1). However, the code psychiatric disorders was more prevalent in the website (difference, 11.2 percentage points) as was investigations, (mostly weight gain) (difference, 7.7 percentage points).

## Discussion

This qualitative study ascertained the type of medication change (discontinuation, dose change, or medication switch) and the reasons for the change. While adverse events predominated as the reason for a medication change, other reasons were apparent, such as medication costs and medication being perceived as unnecessary.

We compared WebMD data with spontaneous reporting systems. Although those reporting systems do not track medication changes, it can be assumed that most reported adverse events were bothersome enough to discuss with a health care professional. Our ranking comparisons of adverse events for individual SSRIs reported to FAERS and the MHRA demonstrated many similarities, such as a high frequency of psychiatric disorders. However, the people posting on the health website were more likely to report weight gain (under the SOC code investigations) and sexual dysfunction or loss of libido (under the SOC codes reproductive systems and breast disorders and psychiatric disorders). Reports from the health website may reflect adverse events that are more likely to lead to medication discontinuation or that may not be reported as commonly to regulatory agencies due to, for example, patient embarrasment^29^ or the adverse event being deemed so well-known that another report is thought not to be worthwhile.

When considering percentage differences in adverse events, the code injury, poisoning, and procedural complications appeared more frequently in FAERS data than in data from the health website. On closer inspection of the FAERS data, this category was commonly used for toxic effects, overdose (intentional and accidental), misuse, and exposure during pregnancy—categories unlikely to be frequently posted on a drug review platform. Although the code psychiatric disorders was highly ranked in the website, FAERS, and MHRA data, the proportion of these adverse events that was cited was higher in the website. It is challenging to study adverse events that are similar to the treated indication. However, further investigation as to whether these adverse events lead to higher discontinuation is warranted. This research could ascertain whether the higher proportion of psychiatric disorders in website posts reflects a higher rate of reporting or whether these types of adverse events are more salient to decisions to discontinue an SSRI. The higher proportion of investigations listed in the website than in MHRA as noted earlier may reflect reporting rates or may similarly be adverse events that lead to discontinuation.

A direct comparison of the reasons leading to SSRI discontinuation found in our study with the reasons identified in the literature is not possible given that the sources of data have inherent differences; however, many similarities were identified. Previous research has identified several factors contributing to SSRI nonadherence, including physician-related issues (education, communication, and prescription patterns), patient-related issues (motivation, cost, and perception of treatment), and medication-related issues (adverse event profile, delayed onset, and titration schedules).^4^ Our study found reasons for medication changes in all 3 of these categories. Similar descriptions of adverse events from previous studies were reported as reasons for medication change in our study, and concerns about medication cost and the need of the SSRI were also found in our data.^4,12^ Notably, reports of people discontinuing SSRIs without the guidance of a clinician were also found in our data, affirming the need to improve prescriber communication and trust.

Beyond the expressed immediate reason for medication treatment change, the contextual information gleaned from patients through online drug reviews may help inform interventions to improve SSRI persistence.^30,31,32^ Interventions focusing on patient-centric elements, including potential adverse events, may improve medication adherence.^32^ However, reading through a list of potential adverse events does not always have the intended effect. Indeed, a drug pamphlet may increase nocebo effects. The nocebo effect is a situation in which patients develop adverse effects or symptoms that can occur with a drug or other therapy merely because the patients believe that they may experience them. Patient-centered communication about adverse effects could be achieved by informing them of the nocebo effect or by framing adverse effects as onset sensations of the medication. Information from an online forum could also be used to tailor the clinician-patient interaction with recommendations that echo the patient’s own perspective, enabling health care professionals to engage more fully with their patients.^30^ Careful consideration of the importance placed on different adverse events and individual patient risk factors prior to initiating pharmacotherapy may lead to selection of the most appropriate individualized treatment choices, which may improve adherence.^33^ Many of the posts reported discontinuation of SSRIs after experiencing an adverse event shortly after initiating their medication. Quick follow-up, for instance, within the first 2 weeks of beginning a new SSRI, may be useful to identify patients who discontinue early without the guidance of a professional.

### Limitations

An important limitation of our study is that it may not represent SSRI recipients in general. The majority of our sample was female, but this might reflect higher SSRI use among female patients.^34^ The mean ages of initial prescription of an SSRI are 39 years in the UK^35^ and 47 years in the US.^36^ Individuals in our sample, therefore, may be slightly younger than those mean ages and may reflect the age of people posting on the health-related website.^37^

We could not validate every review or ensure that 1 reviewer did not create multiple accounts to lower the rating of certain medications, perhaps out of personal frustration or anti–pharmaceutical corporation sentiments. However, we included only drug reviews with commentaries for study, and each commentary was unique and often quite detailed or lengthy, making such sabotage unlikely.

Although the website we studied is commonly used, the number of drug reviews posted has decreased in recent years (which may be because people look for similar reviews before they post), and it does not represent people who do not post on social media or who prefer other online forums. Future research could investigate a wider range of social media platforms, including Twitter, Reddit, and patient forums.^38^ This may require more complex data extraction methods in generic platforms outside the drug review arena.

Our limited sample size prevented us from investigating the association of key demographic characteristics with reasons for medication change. For example, we were unable to identify whether older and longer-term recipients experienced fewer emotional and behavioral adverse events, as suggested in the literature.^37^ Specific adverse events are known to vary across SSRI medications, creating different adverse event profiles,^20,33,39,40,41,42^ while efficacy has shown no clinically important differences.^43^ With a larger sample size, we could have explored this more fully. Comparison with spontaneous reporting systems is not ideal due to differences in purpose and collection methods. However, both spontaneous reporting systems and social media may overestimate the relative frequency of adverse events that are more likely to be reported.^20,44^

## Conclusions

The findings of this qualitative study suggest that it is not merely feasible to collect data from online comments and reviews regarding SSRI medication changes, but that doing so can provide important supplementary information to reporting systems. These data can be broken down by type of change, type of medication, and reason for change, including named adverse events, and are valuable because they reflect people’s behaviors and motivations to change medication. Future research on a larger scale could be used to examine adverse event profiles with different antidepressants and stratification by key demographic characteristics.

## Notes

Supplement 1. **eAppendix.** Annotation Guidelines **eTable 1.** Condition for Which SSRIs Were Prescribed Selected From a Drop-Down Menu **eTable 2.** Reasons for Discontinuation, Dose Change or SSRI Switching **eTable 3.** Example Reviews From WebMD Regarding Health Professional Involvement **eTable 4.** FDA Data for SSRIs **eTable 5.** Mentions of Adverse Events by MedDRA SOC Code in FDA and WebMD **eTable 6.** MHRA Data for SSRIs **eTable 7.** Mentions of Adverse Events by MedDRA SOC Code in MHRA and WebMD **eFigure 1.** Number of People Posting Within Each Age Category **eFigure 2.** Number of People Posting Within Each Category of Duration of SSRI Medication Use

Supplement 2.
Data Sharing Statement
