# Supplementary material for: Reasons for Discontinuation or Change of Selective Serotonin Reuptake Inhibitors in Online Drug Reviews
Source: JAMA Netw Open. 2023 Jul 17;6(7):e2323746. doi: 10.1001/jamanetworkopen.2023.23746 (PMC10352861; doi:10.1001/jamanetworkopen.2023.23746)
Supplement: Supplement 1. — eAppendix. Annotation Guidelines eTable 1. Condition for Which SSRIs Were Prescribed Selected From a Drop-Down Menu eTable 2. Reasons for Discontinuation, Dose Change or SSRI Switching eTable 3. Example Reviews From WebMD Regarding Health Professional Involvement eTable 4. FDA Data for SSRIs eTable 5. Mentions of Adverse Events by MedDRA SOC Code in FDA and WebMD eTable 6. MHRA Data for SSRIs eTable 7. Mentions of Adverse Events by MedDRA SOC Code in MHRA and WebMD eFigure 1. Number of People Posting Within Each Age Category eFigure 2. Number of People Posting Within Each Category of Duration of SSRI Medication Use [file jamanetwopen-e2323746-s001.pdf]

Supplementary Online Content

Golder S, Medaglio D, O’Connor K, Hennessy S, Gross R, Gonzalez Hernandez G. Reasons for discontinuation or change of selective serotonin reuptake inhibitors in online drug reviews. *JAMA Netw Open*. 2023;6(7):e2323746. doi:10.1001/jamanetworkopen.2023.23746

eAppendix. Annotation Guidelines

eTable 1. Condition for Which SSRIs Were Prescribed Selected From a Drop-Down Menu

eTable 2. Reasons for Discontinuation, Dose Change or SSRI Switching

eTable 3. Example Reviews From WebMD Regarding Health Professional Involvement

eTable 4. FDA Data for SSRIs

eTable 5. Mentions of Adverse Events by MedDRA SOC Code in FDA and WebMD

eTable 6. MHRA Data for SSRIs

eTable 7. Mentions of Adverse Events by MedDRA SOC Code in MHRA and WebMD

eFigure 1. Number of People Posting Within Each Age Category

eFigure 2. Number of People Posting Within Each Category Of Duration of SSRI Medication Use

This supplementary material has been provided by the authors to give readers additional information about their work.

© 2023 Golder S et al. *JAMA Network Open*.

eAppendix. Annotation Guidelines

Project Background:

Medication nonadherence refers to when a patient does not adhere to their prescription regimen. Nonadherence to antidepressants can have severe consequences (including withdrawal syndrome and relapse), and it has become a serious issue as over 50% discontinue their medication within the first 3 months. Researching and monitoring the reasons behind antidepressant medication non-adherence is essential to finding an effective solution for it. In this project, we will be manually annotating a set of WebMD comments for Selective Serotonin Reuptake Inhibitors that indicate a medication change.

General Annotation Instructions

1. Medication change column: if the comment does not mention a medication change or mentions a medication change that may happen in the future, it should be coded with a '0'. If the comment mentions a medication change that has already happened or will happen that day, it should be coded with a 'd' for discontinuation (use if there is no mention of a subsequent drug being initiated), 's' for medication switch (include brand/generic switches), or 'c' for a dosage change (includes dose titrations).
2. Health care consultation column: coded with 'yes', 'no', or 'unsure' depending on whether the author appears to have had healthcare direction to make the medication change indicated above.
3. Reason for change columns: one or two reasons from the dropdown list of reasons for the change should be selected for each comment that is a positive for medication change- 1: adverse effects, 2: cost/insurance, 3: drug not working (to be used with discontinuations and drug switches), 4: dose titration (to be used to further classify a dose change), 5: not necessary, 6: unsure, 7: other
4. Adverse effects extraction: if one of the reasons for nonadherence in the previous column was 'adverse effects', then the annotator should extract the mentions of specific adverse effects from the comment and insert them into this column. Do not use this to describe withdrawal syndrome.
5. Withdrawal Syndrome: If the review contains a description of adverse effects that occurred as the drug was tapered down and/or discontinued, code as 'yes'. Else, 'no'.
6. Days to adverse effects: if mentioned, amount of time (in days) after start of medication regimen at which adverse effects started should be recorded in this column. Do not use to describe days to withdrawal syndrome effects.
7. Rechallenge and "Side Effects back?" columns: if comment mentions retrying the medication after a discontinuation, the rechallenge column should be coded with a 'yes'; "side effects back?" column should be coded with a 'yes' or a 'no' depending on whether adverse effects return after restarting certain medication
8. Notes column: any additional interesting or recurring details mentioned in the comment should be noted in this column

© 2023 Golder S et al. *JAMA Network Open*.

What should be coded as a medication change?

1. Any mention of a change in medication regimen that has already occurred or will occur that day (a discontinue, increase/decrease in dose, or change of medication)
2. Mentioned medication change should relate to a personal experience of the author

What should be coded as an adverse effect?

1. Effects originate from taking the specific drug the comment was made for, and they were not due to taking too much of the drug
2. Author personally experiences the effects
3. Effect can be coded to a medical term in the dropdown menu even if it is in colloquial terms

Additional Documentation

Many reviews detail experiences on a different SSRI, yet are submitted under a different SSRI. Create a new line to document the SSRI described in the review. Indicate that this information on a different SSRI was found in a review by marking "Additional SSRI discussed" as 'yes'

EXAMPLE 1: *Paxil (paroxetine)*

"used it 5 or so years. worked well, no side effects. getting off it has been a nightmare.(small joke). even cutting down by 5mg increments every 6-8 weeks produces faintness, trouble falling asleep, the whole host of "discontinuation symptoms" (aka withdrawls). Didn't know anything about this aspect until I tried to stop cold turkey- Dr said it wouldn't be a problem. My advice- try everything before SSRI's.."

Medication Change	Health care consultation	Reason for change	Adverse effects extraction	Withdrawal syndrome	Days to adverse effect	Rechallenge	Side effects back?
d	yes	6		yes			

EXAMPLE 2: *sertraline*

"I began taking a low dose of sertraline in 2011 after years and years with no treatment for my depression, anxiety, PTSD and panic. I thought I'd gotten better and stopped taking it (please, never do that) and got really worse. I am back on, now taking 100mg in the morning and 100mg at night and I feel much better again, like my old self. This medication has quite possible saved my life."

Medication Change	Health care consultation	Reason for change	Adverse effects extraction	Withdrawal syndrome	Days to adverse effect	Rechallenge	Side effects back?
d	no	5		no		yes	

© 2023 Golder S et al. *JAMA Network Open*.

eTable 1. Condition for Which SSRIs Were Prescribed Selected From A Drop-down Menu

Condition from drop-down menu	Number of Reviews (n=667)
Depression	240
Major Depressive Disorder	105
Panic Disorder	68
Other	65
Repeated Episodes of Anxiety	55
Obsessive Compulsive Disorder	53
Bipolar Depression	34
Posttraumatic Stress Syndrome	13
"Change of Life" Signs	13
Extreme Apprehension or Fear of Social Interaction	7
Anxiousness associated with Depression	7
Premenstrual Disorder with a State of Unhappiness	2
Bulimia	1
Depression following Delivery of Baby	1
Disorder characterized by Stiff, Tender & Painful Muscles	1
Frequent Headaches	1
Muscle Weakness associated with Sleeping Disease	1

© 2023 Golder S et al. *JAMA Network Open*.

eTable 2. Reasons for Discontinuation, Dose Change or SSRI Switching

	Adverse events	Titration	Not working	Unclear	Not necessary	Other (such as pregnancy, ran out)
Discontinuation	231	0	31	47	20	9
Dose Change	28	143	5	8	5	2
Switching SSRI	87	0	62	25	0	2
Total	346	143	98	80	25	13

NB: For 24 changes more than one reason was given, most commonly adverse events and not working (21 instances).

© 2023 Golder S et al. *JAMA Network Open*.

eTable 3. Example Reviews From WebMD Regarding Health Professional Involvement

Discontinuation because no longer felt medication needed	*I no longer felt I needed this medication so I took myself off it. Going off the medication was very hard, but I had to just made up my mind whatever my doctor said that I was going to do it. I kept cutting my dosage in half, taking the new dose for a week at a time until I was down to a quarter of a pill and then I stopped altogether. (paraphrased from female patient 45-54 years old on SSRI 10 years or more)*
Discontinuation because of adverse events	*It is happening so fast, and I have only been on this medication for about a week now. I stayed in bed all day today as I just could not do a thing. If that is what this drug is going to do to me, make me not care about anything, then I am going to quit taking it. It is a good thing I only took 1/2 a pill each day instead of what my psychiatrist told me to take. I would hate to see how much worse It would have been had I taken the whole dose each day. (paraphrased from female patient 35-44 years old on SSRI less than one month)*
Discontinuation because of health insurance	*My son couldn't get this medication on his low cost drug plan and ran out completely. He is unemployed and has no health insurance. Going cold turkey was horrible for him. The mental health clinic gave him a few more samples, but I worry what will happen when he runs out again. (paraphrased from female caregiver))*
Discontinuation because of availability and adverse events	*I took this medication for 8 months. After about 1 month, I slept all day long everyday and could hardly function with no energy or interest in anything. On Thursday after Christmas, I realized I had run out of pills. I could not be bothered to refill my prescription or pick it up. On the Wednesday six days off my medication, I woke up feeling like a cloud had lifted; I felt so much better. I looked up users reviews and found that extreme fatigue and lack of motivation that I had are common, but it isn't on the drug information leaflet and my prescriber said she wasn't aware. (I cannot understand why didn't she check into it when I was so bad for so long). I am not going back on this ever (paraphrased from female patient 55-64 years old on SSRI 6 months to less than 1 year)*

© 2023 Golder S et al. *JAMA Network Open*.

eTable 4. FDA Data for SSRIs

All adverse events							
	Escitalopram	Sertraline	Citalopram	Paroxetine	Fluvoxamine	Vortioxetine	Fluoxetine
**Number of cases**	33,150	92,010	28,277	67,869	6,767	13,076	86,169
**Male**	32% (10666/33150)	31% (28464/92010)	34% (9557/28277)	32% (21723/67869)	40% (2686/6767)	26% (3437/13076)	27% (23522/86169)
**Female**	58% (19250/33150)	58% (53107/92010)	55% (15416/28277)	58% (39487/67869)	46% (3139/6767)	61% (7937/13076)	58% (49931/86169)
**Unknown gender**	10% (3234/33150)	11% (10439/92010)	12% (3304/28277)	10% (6659/67869)	14% (942/6767)	13% (1702/13076)	15% (12716/86169)
**1st SOC Ranking**	Psychiatric disorders	Psychiatric disorders	Psychiatric disorders	General disorders and administration site conditions	Nervous system disorders	Psychiatric disorders	Psychiatric disorders)
**2nd SOC Ranking**	General disorders and administration site conditions	General disorders and administration site conditions	Injury, poisoning and procedural complications	Psychiatric disorders	Psychiatric disorders	General disorders and administration site conditions	Nervous system disorders
**3rd SOC Ranking**	Nervous system disorders	Nervous system disorders	Nervous system disorders	Nervous system disorders	General disorders and administration site conditions	Gastrointestinal disorders	General disorders and administration site conditions
**4th SOC Ranking**	Injury, poisoning and procedural complications	Injury, poisoning and procedural complications	General disorders and administration site conditions	Injury, poisoning and procedural complications	Investigations	Nervous system disorders	Injury, poisoning and procedural complications)

© 2023 Golder S et al. *JAMA Network Open*.

eTable 5. Mentions of Adverse Events by MedDRA SOC Code in FDA and WebMD

	% of FDA mentions							FDA Total N=737798	**WebMD N=968**	Difference FDA and WebMD
	Escitalopram	Sertraline	Citalopram	Paroxetine	Fluvoxamine	Vortioxetine	Fluoxetine			
Psychiatric disorders	16.3	17.3	16.9	16.8	16.8	18.6	20.1	17.7	**35.0**	17.3 (13.6, 20.3)
Nervous system disorders	15.2	15.1	13.9	15.9	17.2	13.9	14.4	15.0	**16.6**	1.6 (-0.7, 3.9)
Gastrointestinal disorders	7.0	8.6	6.4	7.4	7.6	13.9	6.4	7.5	**12.8**	5.3 (3.2, 7.4)
Investigations	7.1	6.0	6.9	5.9	9.2	5.6	6.3	6.4	**10.6**	4.2 (2.3, 6.1)
General disorders and administration site conditions	15.4	16.0	13.6	18.6	15.0	15.4	14.3	15.7	**8.2**	-7.5 (-9.2, -5.8)
Skin and subcutaneous tissue disorders	3.6	4.4	3.5	4.2	4.3	7.5	5.5	4.5	**5.2**	0.7 (-0.6, 2.1)
Reproductive system and breast disorders	1.4	2.0	1.4	1.5	1.4	1.4	1.9	1.7	**4.0**	2.3 (1.1, 3.5)
Metabolism and nutrition disorders	4.0	3.7	4.3	3.4	3.3	2.7	3.5	3.7	**2.2**	1.5 (0.3, 2.7)
Musculoskeletal and connective tissue disorders	3.5	3.8	3.4	3.1	3.4	2.9	3.6	3.5	**1.3**	-2.2 (-2.9, -1.5)
Respiratory, thoracic and mediastinal disorders	4.1	4.1	4.8	3.5	4.1	1.9	4.0	4.0	**1.1**	-2.9 (-3.6 -2.2)
Cardiac disorders	4.8	3.8	5.8	3.7	4.1	2.0	4.4	4.3	**0.8**	-3.5 (-4.1, -2.9)
Eye disorder	2.1	2.3	2.0	2.3	2.3	2.5	2.0	2.2	**1.2**	-1.0 (-1.7, -0.3)
Renal and urinary disorders	1.8	1.7	1.8	1.4	2.2	1.2	1.7	1.6	**0.5**	-1.1 (-1.5, -0.7)
Ear and labyrinth disorders	0.9	1.0	0.7	1.7	0.5	1.1	0.7	1.0	**0.2**	-0.8 (-1.1, -0.5)
Injury, poisoning and procedural complications	12.6	10.1	14.6	10.6	8.6	9.3	11.2	11.3	**0.1**	-11.2 (-11.4, -11.0)

© 2023 Golder S et al. *JAMA Network Open*.

eTable 6. MHRA Data for SSRIs

All adverse events							
	Escitalopram	Sertraline	Citalopram	Paroxetine	Fluvoxamine	Vortioxetine	Fluoxetine
**Number of reports**	529	4790	3556	1416	29	317	2158
**Number of AEs**	1806	13903	10896	12853	112	693	6683
**Male**	31% (166/529)	30% (1449/4790)	33% (1180/3556)	36% (514/1416)	55% (16/29)	34% (109/317)	31% (669/2158)
**Female**	62% (326/529)	62% (2946/4790)	63% (2255/3556)	60% (849/1416)	45% (13/29)	62% (197/317)	64% (1384/2158)
**Unknown gender**	7% (37/529)	3% (160/4790)	3% (121/3556)	4% (53/1416)	0% (0/29)	3% (11/317)	5% (105/2158)
**1st SOC Ranking**	Psychiatric disorders	Psychiatric disorders	Psychiatric disorders	Psychiatric disorders	Gastrointestinal disorders	Psychiatric disorders	Nervous system disorders
**2nd SOC Ranking**	Nervous system disorders	Nervous system disorders	Nervous system disorders	Nervous system disorders	Nervous system disorders	Gastrointestinal disorders	Psychiatric disorders
**3rd SOC Ranking**	General disorders and administration site conditions	Gastrointestinal disorders	Gastrointestinal disorders	General disorders and administration site conditions	Psychiatric disorders	Nervous system disorders	Skin and subcutaneous tissue disorder
**4th SOC Ranking**	Gastrointestinal disorders	General disorders and administration site conditions	General disorders and administration site conditions	Gastrointestinal disorders	General disorders and administration site conditions	General disorders and administration site conditions	Gastrointestinal disorders

© 2023 Golder S et al. *JAMA Network Open*.

eTable 7. Mentions of Adverse Events by MedDRA SOC Code in MHRA and WebMD

	% of MHRA mentions							Total MHRA N=100098	WEBMD N=968	Difference Total MHRA and WebMD
	Escitalopram	Sertraline	Citalopram	Paroxetine	Fluvoxamine	Vortioxetine	Fluoxetine			
Psychiatric disorders	26.9	21.4	22.0	28.1	14.2	25.2	21.6	23.8	**35.0**	11.2 (8.2, 14.2)
Nervous system disorders	20.0	18.6	19.3	22.7	23.2	16.3	19.0	20.5	**16.6**	-3.9 (-6.3, -1.5)
Gastrointestinal disorders	11.4	15.2	11.3	10.8	35.1	22.5	12.7	13.4	**12.8**	-0.6 (-2.7, 1.5)
Investigations	3.5	3.4	4.5	2.0	1.3	2.2	3.3	2.9	**10.6**	7.7 (5.8, 9.6)
General disorders and administration site conditions	11.4	10.8	10.8	15.2	9.9	9.9	9.4	12.0	**8.2**	-3.8 (-5.5,-2.1)
Skin and subcutaneous tissue disorders	6.4	7.7	6.9	5.9	4.8	7.8	12.9	7.9	**5.2**	-2.7 (-4.1, -1.3)
Reproductive system and breast disorders	2.5	2.7	2.5	2.2	1.0	2.6	2.5	2.3	**4.0**	1.7 (0.5, 2.9)
Metabolism and nutrition disorders	1.9	3.4	4.4	1.9	2.1	2.1	3.6	2.9	**2.2**	0.7 (-0.3, 1.8)
Musculoskeletal and connective tissue disorders	3.2	3.5	3.3	2.5	1.9	3.0	3.8	3.1	**1.3**	-1.8 (-2.5, -1.1)
Respiratory, thoracic and mediastinal disorders	2.6	3.0	3.2	1.2	0.8	1.0	2.9	2.2	**1.1**	-1.1 (-1.7, -0.4)
Cardiac disorders	2.3	2.1	3.0	1.5	2.2	1.0	2.1	2.0	**0.8**	-1.2 (-1.8, -0.6)
Eye disorder	2.4	3.0	2.6	2.4	1.6	1.5	2.1	2.5	**1.2**	-1.3 (-2.0, -0.6)
Renal and urinary disorders	1.3	1.3	1.4	0.7	1.0	1.0	1.1	1.0	**0.5**	-0.5 (-0.9, 0.1)
Ear and labyrinth disorders	0.7	1.0	1.0	1.3	0.6	0.5	0.6	1.0	**0.2**	-0.8 (-1.1, -0.5)
Injury, poisoning and procedural complications	3.5	3.1	3.9	1.7	0.4	3.5	2.5	2.5	**0.1**	-2.4 (-2.6, -2.2)

© 2023 Golder S et al. *JAMA Network Open*.

eFigure 1. Number of People Posting Within Each Age Category

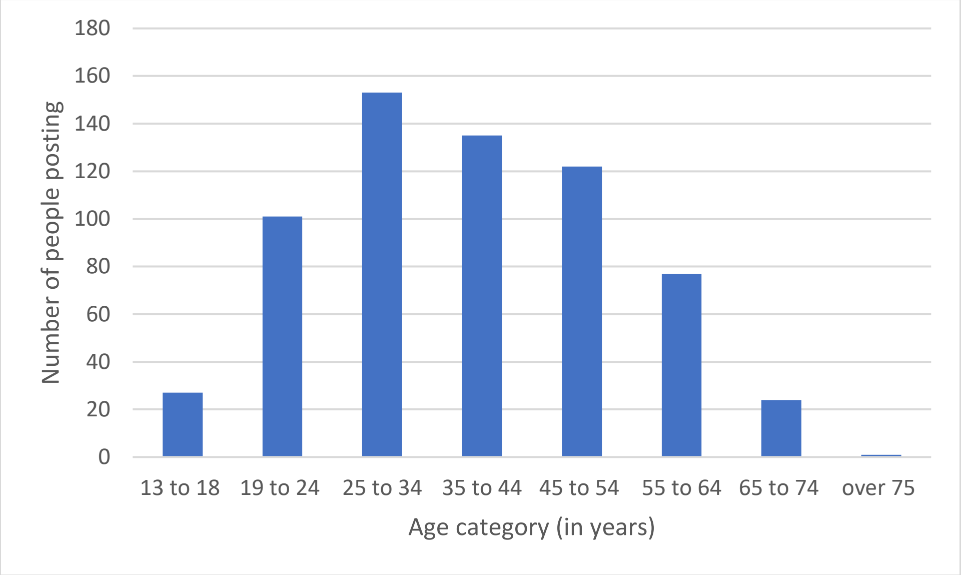

NB: 19 people did not record their age category

© 2023 Golder S et al. *JAMA Network Open*.

eFigure 2. Number of People Posting Within Each Category Of Duration of SSRI Medication Use

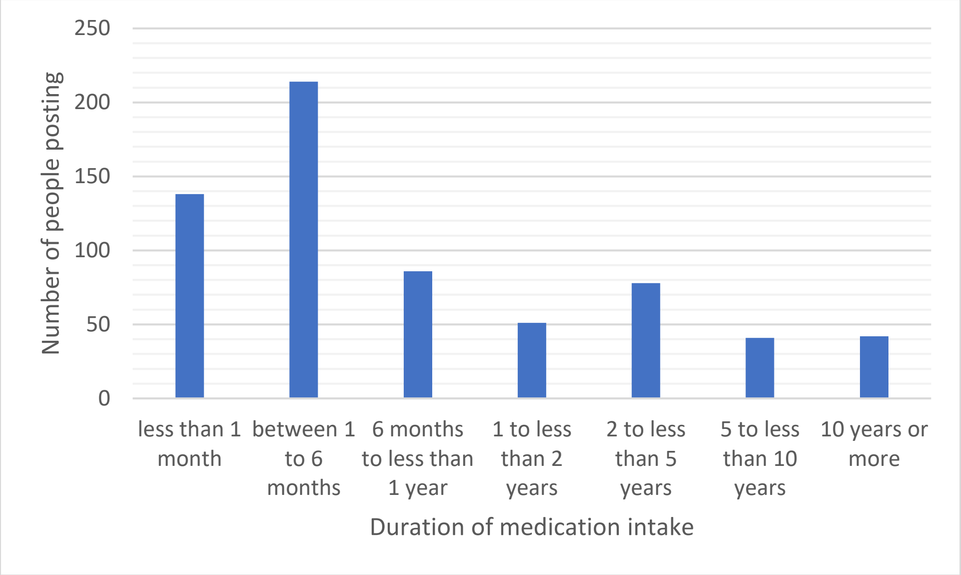

NB: In 17 reviews the time on the medication was not given.

© 2023 Golder S et al. *JAMA Network Open*.
