# Supplementary material for: Reasons for Discontinuation or Change of Selective Serotonin Reuptake Inhibitors in Online Drug Reviews
Source: JAMA Netw Open. 2023 Jul 17;6(7):e2323746. doi: 10.1001/jamanetworkopen.2023.23746 (PMC10352861; doi:10.1001/jamanetworkopen.2023.23746)
Supplement: Supplement 2. — Data Sharing Statement [file jamanetwopen-e2323746-s002.pdf]

Data Sharing Statement

Golder. Reasons for Discontinuation or Change of Selective Serotonin Reuptake Inhibitors in Online Drug Reviews. *JAMA Netw Open*. Published July 17, 2023.
doi:10.1001/jamanetworkopen.2023.23746

Data
Data available: No

Additional Information
Explanation for why data not available: The WebMD drug reviews are already available online on the WebMD website. We would be violating the WebMD terms and conditions to reproduce them elsewhere.
